# Family History of Mental and Neurological Disorders and Risk of Autism

**DOI:** 10.1001/jamanetworkopen.2019.0154

**Published:** 2019-03-01

**Authors:** Sherlly Xie, Håkan Karlsson, Christina Dalman, Linnea Widman, Dheeraj Rai, Renee M. Gardner, Cecilia Magnusson, Diana E. Schendel, Craig J. Newschaffer, Brian K. Lee

**Affiliations:** 1 Drexel University School of Public Health, Philadelphia, Pennsylvania; 2 Department of Neuroscience, Karolinska Institutet, Stockholm, Sweden; 3 Department of Public Health Sciences, Karolinska Institutet, Stockholm, Sweden; 4 Centre for Epidemiology and Community Medicine, Stockholm County Council, Stockholm, Sweden; 5 Centre for Academic Mental Health, Bristol Medical School, University of Bristol, Bristol, United Kingdom; 6 Avon and Wiltshire Partnership National Health Service Mental Health Trust, Bath, United Kingdom; 7 Department of Public Health, University of Aarhus, Aarhus, Denmark; 8 Department of Economics and Business Economics, University of Aarhus, Aarhus, Denmark; 9 Lundbeck Foundation Initiative for Integrative Psychiatric Research, University of Aarhus, Aarhus, Denmark; 10 A. J. Drexel Autism Institute, Philadelphia, Pennsylvania

## Abstract

**Importance:**

Familial aggregation of mental and neurological disorders is often observed in autism spectrum disorders (ASD), but reports have generally focused on single disorders and are limited to first-degree relatives.

**Objectives:**

To examine family history of mental and neurological disorders among first- to fourth-degree relatives and risk of ASD with and without intellectual disability (ID) in index persons.

**Design, Setting, and Participants:**

In this population-based cohort study, 567 436 index persons were identified from the Stockholm Youth Cohort, an ongoing longitudinal register-linkage cohort study of the total population aged 0 to 17 years residing in Stockholm County, Sweden. Index persons were nonadopted singleton births born between 1984 and 2009 who were at least 2 years of age at the end of follow-up on December 31, 2011, had resided in Stockholm County for at least 2 years since birth, and could be linked to both biological parents. Data analysis was conducted from May 2017 to December 2018.

**Exposure:**

Mental and neurological diagnoses of relatives of the index persons.

**Main Outcomes and Measures:**

Diagnosis of ASD, with or without co-occurring ID, in the index persons.

**Results:**

The cohort included 567 436 index persons (291 191 [51.3%] male; mean [SD] age at the end of follow-up, 14.3 [7.5] years). The prevalence of ASD with and without ID was 0.4% and 1.5%, respectively. Positive family history of mental and neurological disorders was associated with higher odds of ASD in index persons; 6895 (63.1%) of index persons with ASD had a parent with history of mental and/or neurological disorders, compared with 252 454 (45.4%) of index persons without ASD. Family history of multiple disorders was associated with higher odds of ASD in index persons, including history of ASD (odds ratio among first-degree relatives for ASD with and without ID: 14.2, 9.0), intellectual disability (7.6, 2.3), attention-deficit/hyperactivity disorder (3.3, 4.7), obsessive compulsive disorder (1.9, 2.1), schizophrenia and other nonaffective psychotic disorders (2.1, 1.8), depression (1.4, 2.0), bipolar disorder (1.4, 2.2), personality disorder (2.1, 2.6), cerebral palsy (2.2, 1.5), and epilepsy (2.0, 1.3). The more closely related the affected family member was, the higher the odds was of ASD for the index person. ASD without intellectual disability was associated with more disorders compared to ASD with intellectual disability. ASD with intellectual disability exhibited a weaker familial association with other mental disorder diagnoses but a stronger familial association with some neurological diagnoses as compared to ASD without intellectual disability.

**Conclusions and Relevance:**

This study suggests that family history of mental and neurological disorders is associated with increased risk of ASD. The familial component of ASD etiology may differ by presence or absence of co-occurring ID.

## Key Points

**Question:**

Is family history of mental and neurological disorders associated with risk of autism spectrum disorders, and does this vary with vs without intellectual disability?

**Findings:**

In this population-based cohort study of 567 436 Swedish participants, positive family history was associated with increased risk of autism spectrum disorders. Autism spectrum disorders with intellectual disability exhibited a weaker familial association with other mental disorder diagnoses but a stronger familial association with some neurological diagnoses as compared with autism spectrum disorders without intellectual disability.

**Meaning:**

This study suggests that family history of mental and neurological disorders is associated with autism risk, and the familial component of autism etiology may differ by presence or absence of co-occurring intellectual disability.

## Teaser

This population-based cohort study examines family history of mental and neurological disorders among first- to fourth-degree relatives and risk of autism spectrum disorders with and without intellectual disability in index persons from the Stockholm Youth Cohort in Sweden.

## Introduction

Autism spectrum disorders (ASD) are a group of heritable, complex, early-onset neurodevelopmental disorders that affect at least 1% to 2% of the population worldwide.^1,2^ The typical age at diagnosis is approximately 8 years and may vary by sex, co-occurring conditions, and other factors.^3,4^ Approximately 25% of individuals with ASD are also affected by intellectual disability (ID), ie, IQ score less than 70 points.^2^ Individuals with ASD and ID display fewer positive verbal and nonverbal social skills and more challenging behaviors than those with ID alone.^5,6,7^ Studies in clinical collections such as the Simons Simplex Collection suggest that ASD with and without ID may have different familial risk, such that family history of depression, bipolar disorder, or schizophrenia is more relevant in ASD without ID than in ASD with ID.^8^ However, to our knowledge, this has not been examined for a broader spectrum of disorders and separately for different family members.

Family history of ASD recurrence is by far the strongest known risk factor for ASD. For example, twin studies report heritability of ASD in the 60% to 90% range with high concordance rates among monozygotic twins.^9^ However, family history of other mental or neurological disorders may also potentially be associated with ASD risk. Having siblings with ASD, attention-deficit/hyperactivity disorder (ADHD), and ID also has been linked to 11.8-, 3.7-, and 3.1-fold increases in odds of ASD, respectively, in the index person, the individual through whom the family was recruited into the study.^10^ Paternal and maternal histories of schizophrenia and other nonaffective psychotic disorders (NAPD), affective disorders, and childhood-onset disorders have been associated with doubled risk of ASD in offspring.^11^ These studies suggest that a comprehensive, systematically conducted investigation can facilitate identification of both gaps and hot spots in the family history fabric, which cannot be gleaned from studies in which 1 or a few associations are examined. To this end, we conducted a large cohort study on the association between family history of mental and neurological disorders and ASD with and without ID using data from Swedish population registers.

## Methods

### Study Population

We conducted a population-based cohort study in Sweden. Data were collected by linkage of prospectively collected national and regional health and administrative registers using unique personal identification numbers assigned to each Swedish resident. All data were anonymized before being received by the research team. Informed consent was not required for analysis of anonymized register data. Ethical approval was obtained from the regional ethical review board in Stockholm. We followed the Strengthening the Reporting of Observational Studies in Epidemiology (STROBE) reporting guideline.

Index persons were identified from the Stockholm Youth Cohort, an ongoing longitudinal register-linkage cohort study of the total population aged 0 to 17 years residing in Stockholm County, Sweden.^3^ Index persons were nonadopted singleton births born between 1984 and 2009 who were at least 2 years of age at the end of follow-up on December 31, 2011, had resided in Stockholm County for at least 2 years since birth, and could be linked to both biological parents (Figure).^1^ Through the eligible index persons, we ascertained their first- to fourth-degree relatives who had resided in Sweden for at least 2 years. The requirement on minimal length of residence was for ensuring entry of diagnoses into the source registers. The Multi-Generation Register links parents to children for all children born from 1932 onward. This allowed us to determine family relations for each index person up to 4 degrees of relatedness: first-degree relatives with, on average, 50% genetic similarity (GS) included fathers, mothers, and full siblings; second-degree relatives (25% GS) included grandparents, uncles, aunts, and half-siblings; third-degree relatives (12.5% GS) included half-uncles, half-aunts, and first cousins; fourth-degree relatives (6.25% GS) included half-cousins. Follow-up of all participants was confirmed in the Total Population Register and the Cause of Death Register, which contained data on immigration and mortality, respectively.^12,13^

**Figure.**
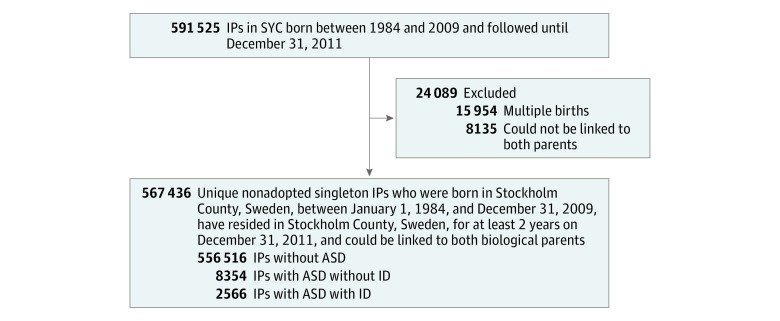
Derivation of IPs From the SYC ASD indicates autism spectrum disorders; ID, intellectual disability; IPs, index persons; and SYC, Stockholm Youth Cohort.

### Ascertainment of Diagnoses

We extracted mental and neurological diagnoses for all index persons and relatives from birth to death or the end of follow-up, whichever came first. Mental disorders included were alcohol misuse, drug misuse, NAPD, bipolar disorder, depression, anxiety disorders, obsessive-compulsive disorder (OCD), stress-related disorders, other neurotic disorders, eating disorder, personality disorder, ID, ADHD, and other childhood disorders. Neurological disorders included were cerebral palsy, epilepsy, multiple sclerosis, migraine, dementia, stroke, and Parkinson disease. These disorders were identified a priori from the literature or shared symptomatic and possibly etiologic overlap with ASD and were available in our data. Diagnoses of ASD were identified in the population registers. Whether or not a concurrent ID diagnosis was made was noted.^3^ Diagnoses of relatives were abstracted using the Swedish version of *International Classification of Diseases* codes (eTable 1 in the Supplement) from the National Patient Register. Validation studies on the diagnoses of ASD^3^ and other disorders including tic, OCD, epilepsy, depression,^14^ bipolar disorder,^15^ and schizophrenia^16^ have reported validity.

### Covariates

Covariates included in the main analysis were birth years of index persons and relatives, as well as sex of the index persons. In the sensitivity analyses, we also considered parental origin of birth, and age of index persons and relatives at the end of follow-up. Data on these variables were extracted from the Total Population Register. All index persons and relatives had complete data on all exposures, outcomes, and covariates.

### Statistical Analysis

We refer to odds ratios (ORs) as the measure of association throughout the article. The outcomes of interest were ASD without ID and ASD with ID in the index person. To examine the strength of association between each outcome and the various diagnoses in relatives, we fitted 1 logistic regression model for each diagnosis in relatives and stratified the analysis by GS. Secular trends were adjusted for using birth years of index persons and relatives. Regression models in the analysis were also adjusted for sex of index persons to account for the male predominance in ASD prevalence. Because of the etiologic overlap and familial aggregation of mental and neurological disorders, we did not adjust for comorbidities or family history of these disorders in the models. Robust standard errors were estimated to account for family clusters identified by index persons.

We conducted a series of sensitivity analyses to address potential biases and test the robustness of our findings. In sensitivity analysis 1, we stratified the analysis by index person’s parental origin of birth (Sweden or non-Sweden) and reestimated ORs to examine whether migration influenced results. In sensitivity analysis 2, we examined whether our findings could have been influenced by immortal time bias. Disorders like ASD typically have childhood onset, while others like Parkinson disease usually occur later in life. The difference between age at the end of follow-up and age at onset for each disorder could induce immortal time bias. In this sensitivity analysis, we reestimated ORs for each diagnosis by excluding individuals whose follow-up ended or death occurred before the diagnosis first became available in our data or before an age at which they could have been reliably diagnosed. Age cutoffs were set at the 0.5th percentile of age at diagnosis unless otherwise noted (eTable 1 in the Supplement). In sensitivity analysis 3, we repeated the analyses while also adjusting for the relatives’ ASD status to rule out confounding by ASD recurrence. In sensitivity analysis 4, we repeated the analysis on earlier-born (1984-1996) vs later-born (1997-2009) index persons. In sensitivity analysis 5, we repeated the analysis on younger vs older relatives of index persons. Younger and older relatives were those who were younger than and at least 40 years of age, respectively, at the end of follow-up or death, whichever came first.

To control for multiple testing, we applied the Benjamini-Hochberg procedure^17^ with a 5% false discovery rate. All analyses were conducted using R, version 3.4.3 (R Foundation).^18^ Robust standard error estimation was implemented using the* rms* package.^19^

## Results

### Description of Study Sample

The cohort included 567 436 unique, nonadopted singleton index persons, of whom 291 191 (51.3%) were male and 276 245 (48.7%) were female. The mean (SD) age at the end of follow-up was 14.3 (7.5) years. The prevalence of ASD with and without ID was 0.4% and 1.5%, respectively. From family linkages, we ascertained 1 859 142 unique pairs of index person and relative at 50% GS, 3 435 173 pairs at 25% GS, 2 746 155 pairs at 12.5% GS, and 613 563 pairs at 6.25% GS. The median (interquartile range) family size of index persons was 15 (10-19) relatives. Index persons with ASD were more likely than those without ASD to have relatives with history of any mental or neurological diagnosis (Table 1, all *P* values from χ^2^ test for equality of proportions were <.001; eTable 2 in the Supplement). The proportion of relatives with positive history also tended to be higher among index persons with ASD without ID vs index persons with ASD and ID (Table 1 and eTable 2 in the Supplement).

**Table 1.** Characteristics of IPs in the Study

Characteristic	No. (%)			
	IPs With ASD (n = 10 920)	IPs With ASD Without ID (n = 8354)	IPs With ASD With ID (n = 2566)	IPs Without ASD (n = 556 516)
Male	7750 (71.0)	5906 (70.7)	1844 (71.9)	283 441 (50.9)
IPs with ≥1 relative with positive history of any mental or neurological diagnosis				
Any relative	10 540 (96.5)	8121 (97.2)	2419 (94.2)	519 231 (93.3)
GS 50% relatives	8060 (73.8)	6211 (74.3)	1849 (72.1)	308 672 (55.5)
Parents	6895 (63.1)	5404 (64.7)	1491 (58.1)	252 454 (45.4)
Full siblings	3724 (34.1)	2761 (43.2)	963 (47.0)	120 128 (21.6)
GS 25% relatives	9385 (85.9)	7394 (93.4)	1991 (89.8)	463 761 (83.3)
Half-siblings	1886 (17.3)	1472 (49.4)	414 (49.9)	59 550 (10.7)
Grandparents	8800 (80.6)	6958 (90.2)	1842 (90.1)	432 625 (77.7)
Uncles or aunts	5363 (49.1)	4231 (59.2)	1132 (61.2)	247 624 (44.5)
GS 12.5% relatives	6093 (55.8)	4824 (67.5)	1269 (69.0)	278 661 (50.1)
Half-uncles or half-aunts	2448 (22.4)	1975 (58.6)	473 (58.5)	100 511 (18.1)
First cousins	4817 (44.1)	3782 (58.0)	1035 (61.1)	220 587 (39.6)
6.25% GS relatives (half–first cousins)	1766 (16.2)	1415 (54.2)	351 (54.0)	67 979 (12.2)

Abbreviations: ASD, autism spectrum disorders; GS, genetic similarity; ID, intellectual disability; IPs, index persons.

### Family History and Odds of ASD Without ID

#### Mental Disorders

Having a first-degree relative with ASD without ID was associated with a 9-fold increase in odds of ASD without ID in index persons compared with those with unaffected first-degree relatives (Table 2; false discovery rate-adjusted *P *values in eTable 3 in the Supplement). In comparison, having a first-degree relative with ASD with ID was associated with a 3.8-fold increase in odds of ASD without ID in index persons. Having a first-degree relative with ADHD, ID, other childhood disorders, alcohol misuse, drug misuse, NAPD, bipolar disorder, depression, anxiety disorders, OCD, stress-related disorders, other neurotic disorders, eating disorder, or personality disorder was associated with 1.5- to 4.7-fold increases in odds of the index person having ASD without ID compared with those with first-degree relatives without each of these conditions. These associations diminished for more distant family relations (Table 2). However, a history of ASD without ID, ADHD, other childhood disorders, or anxiety disorders among fourth-degree relatives remained significantly associated with higher odds of ASD without ID in index persons. For ASD with and without ID, ADHD, and other childhood disorders, the OR for the index persons to develop ASD without ID decreased proportionally to decreasing degree of genetic relatedness. For other diagnoses, such as ID, OCD, and eating disorder, the OR for ASD without ID decreased considerably between first- and second-degree relatives to remain at a low level across third- and fourth-degree relatives (OR, 1.4, 1.1, and 1.1, respectively).

**Table 2.** Risk of Autism Spectrum Disorders With and Without Intellectual Disability in IPs With vs Without a Family History of Mental and Neurological Disorders

Diagnosis in Relatives	OR^a^							
	ASD Without ID in IPs				ASD With ID in IPs			
	GS 50%^b^	GS 25%^c^	GS 12.5%^d^	GS 6.25%^e^	GS 50%^b^	GS 25%^c^	GS 12.5%^d^	GS 6.25%^e^
Mental								
ASD without ID	9.0^f^	2.5^f^	1.8^f^	1.3^g^	4.1^f^	2.3^f^	1.5^f^	1.0
ASD with ID	3.8^f^	2.0^f^	1.5^f^	1.2	14.2^f^	2.8^f^	1.6^h^	0.8
ID	2.3^f^	1.3^f^	1.4^f^	1.4	7.6^f^	2.4^f^	1.3^h^	1.2
ADHD	4.7^f^	1.9^f^	1.5^f^	1.1^g^	3.3^f^	1.6^f^	1.5^f^	1.0
Other childhood disorders	2.9^f^	1.7^f^	1.3^f^	1.2^f^	2.6^f^	1.6^f^	1.3^f^	1.0
Alcohol misuse	1.5^f^	1.2^f^	1.1^f^	1.1	1.2^g^	1.1	1.2^g^	1.2
Drug misuse	1.8^f^	1.3^f^	1.2	1.0	1.4^f^	1.2^g^	1.2^g^	1.1
Schizophrenia or other NAPD	1.8^f^	1.3^f^	1.2^g^	1.0	2.1^f^	1.2^h^	1.2	0.9
Bipolar disorder	2.2^f^	1.3^f^	1.1	1.3	1.4^g^	1.2	0.9	1.1
Depression	2.0^f^	1.2^f^	1.1^f^	1.2	1.4^f^	1.2^f^	1.1^h^	1.0
Anxiety disorders	1.8^f^	1.1^f^	1.2^f^	1.1^f^	1.4^f^	1.2^f^	1.1^h^	1.0
OCD	2.1^f^	1.3	1.1	1.1	1.9^f^	1.5^g^	0.9	0.6
Stress-related disorders	1.8^f^	1.1^f^	1.1^f^	1.6	1.4^f^	1.2	1.1^h^	1.0
Other neurotic disorders	1.6^f^	1.2^f^	1.1	1.1	1.6^f^	1.2^h^	1.3^h^	1.2
Eating disorder	1.5^f^	1.2^f^	1.1^g^	1.1	1.0	0.9	1.2^h^	1.1
Personality disorder	2.6^f^	1.4^f^	1.2^f^	1.4	2.1^f^	1.3	1.2	1.1
Neurological								
Cerebral palsy	1.5^g^	0.9	1.1	1.3	2.2^g^	1.4	0.7	1.0
Epilepsy	1.3^f^	1.1^f^	1.1	1.1	2.0^f^	1.2^h^	0.9	1.5^h^
Multiple sclerosis	1.3	1.0	1.1	1.2	0.9	0.9	1.1	0.7
Migraine	1.3^f^	1.1	1.1	1.0	1.2^h^	1.1	1.0	1.1
Dementia	0.7^h^	1.1^f^	1.1	1.1	1.2	1.1^f^	1.2^h^	1.1
Stroke	0.7	1.0	0.9	1.4	0.5	1.1^h^	1.1	0.8
Parkinson disease	9.1	1.0	1.7	3.6	NA^i^	1.0	1.9	5.5

Abbreviations: ADHD, attention-deficit/hyperactivity disorder; ASD, autism spectrum disorders; GS, genetic similarity; ID, intellectual disability; IPs, index persons; NA, not applicable; NAPD, nonaffective psychotic disorder; OCD, obsessive-compulsive disorder; OR, odds ratio.

^a^ ORs with multiplicity-adjusted *P* values .05 or higher were not marked.

^b^ The GS 50% relatives included fathers, mothers, and full siblings.

^c^ The GS 25% relatives included grandparents, uncles, aunts, and half-siblings.

^d^ The GS 12.5% relatives included half-uncles, half-aunts, and first cousins.

^e^ The GS 6.25% relatives included half-cousins.

^f^ With multiplicity-adjusted *P* values less than .0001.

^g^ With multiplicity-adjusted *P* values .0001 through less than .01.

^h^ With multiplicity-adjusted *P* values .01 through less than .05.

^i^ The OR could not be estimated owing to low event rate.

#### Neurological Disorders

Having a first-degree relative with cerebral palsy, epilepsy, or migraine was associated with increases in odds of ASD without ID in index persons by 1.5-, 1.3-, and 1.3-fold, respectively, compared with those with unaffected first-degree relatives. The ssociation decreased with decreasing degree of relatedness between index persons and their relatives. Having a third-degree relative with each of these disorders was associated with increases in odds of ASD without ID in index persons by 1.1-fold, compared with those with unaffected third-degree relatives (Table 2; adjusted *P* values in eTable 3 in the Supplement).

### Family History and Odds of ASD With ID

Compared with ASD without ID, ASD with ID was associated with a family history of fewer disorders. For most disorders that were associated with both ASD subtypes, effect sizes of the associations for ASD with ID were smaller, except for ASD with ID, ID, NAPD, cerebral palsy, and epilepsy.

#### Mental Disorders

Having a first-degree relative with ASD with ID was associated with a 14.2-fold increase in odds of the same outcome in index persons compared with those with unaffected first-degree relatives (Table 2; adjusted *P* values in eTable 3 in the Supplement). Having a first-degree relative with ASD without ID was associated with a 4.1-fold increase in odds of ASD with ID in index persons. In addition, having a first-degree relative with ADHD, ID, other childhood disorders, alcohol misuse, drug misuse, NAPD, bipolar disorder, depression, anxiety disorders, OCD, stress-related disorders, other neurotic disorders, or personality disorder was associated with ORs as low as 1.2 (for alcohol misuse) and as high as 7.6 (for ID). These associations decreased for more distant family relations, although many of these disorders in third-degree relatives were still associated with higher odds of ASD with ID in index persons. None of the diagnoses among fourth-degree relatives were associated with increased odds of ASD with ID in index persons.

#### Neurological Disorders

Having a first-degree relative affected by cerebral palsy or epilepsy was associated with doubled odds of ASD with ID in index persons. The association decreased with decreasing degree of relatedness between index persons and their relatives. Compared with those with unaffected third-degree relatives, having a third-degree relative with each of these disorders was associated with lower odds of ASD without ID in index persons that were not different from the null (OR: cerebral palsy, 0.7 and epilepsy, 0.9) (Table 2; adjusted *P* values in eTable 3 in the Supplement).

### Sensitivity Analysis

In sensitivity analysis 1, there were no substantial differences between ORs of index persons whose parents were both born in Sweden and that of index persons with at least 1 parent not born in Sweden (eTable 4 in the Supplement). We observed more precise estimates for index persons whose parents were both born in Sweden, especially among second- to fourth-degree relatives. In sensitivity analysis 2, eTable 5 in the Supplement was nearly identical to Table 2, suggesting our estimates were not substantially affected by immortal time bias. In sensitivity analysis 3, adjusting for the relatives’ ASD status, magnitudes of most ORs decreased slightly but the overall trend was similar to that of Table 2 (eTable 6 in the Supplement). In sensitivity analysis 4, there were no substantial differences between OR estimates on earlier- vs later-born index persons (eTable 7 in the Supplement). Results on earlier-born index persons were more precise. In sensitivity analysis 5, there were no substantial differences between ORs estimated using data from older vs younger relatives of index persons (eTable 8 in the Supplement). Results on younger relatives were more precise.

## Discussion

This study examined the association between family history of mental and neurological disorders and ASD with and without ID. Principal findings from this study are (1) family history of multiple mental and neurological disorders was associated with increased odds of both ASD with and without ID; (2) consistent with the hypothesis that these associations are primarily driven by shared-genetic liability, the more closely related the family member with the mental or neurological disorder was, the greater the risk of ASD; and (3) ASD without ID was associated with a broader spectrum of disorders, and the association was greater than with ASD with ID, consistent with the hypothesis that ASD without ID is more familial than ASD with ID.

To our knowledge, this is the largest study to date on this topic in terms of sample size and number of disorders examined, the first one that investigated ASD subtyped by intellectual ability, and one of few that quantified the association between family mental and/or neurological history and ASD beyond first-degree kinships. Since both ASD subtypes are linked to a number of overlapping mental and neurological disorders, findings from this study agree with previous findings on parents,^11^ siblings,^10^ and first-degree relatives overall^20^ for ASD, regardless of presence or absence of co-occurring ID. For example, Jokiranta-Olkoniemi et al^10^ reported increased risk ratio of ASD without ID in index persons with siblings affected by ASD, ADHD, ID, NAPD and other neurotic, and personality disorders; these disorders were also linked to increased odds of ASD with ID in the index persons in our study.

In addition to recent large-scale genetics studies quantifying the extent of shared heritability in common brain disorders,^21,22,23,24^ findings of this study contribute to a better understanding of family history in ASD. For example, Anttila et al^21^ reported that cognitive performance in early life was associated with the genetic risk for both psychiatric and neurological brain disorders based on genome-wide association studies results on more than 1 million individuals.^23^ Grove et al^22^ identified overlapping genetic architectures between ASD, schizophrenia, major depression, and education attainment and observed substantial differences between single-nucleotide polymorphism–based heritability of ASD subtypes, with ASD without ID being more heritable than ASD with ID. Large dynamic triplet expansions of CGG repeats of the fragile X mental retardation 1 gene on the X chromosome are heritable and cause fragile X syndrome, which occurs in 2% to 8% of ASD cases and in 1% to 2% of ID cases.^25,26,27^ An increasing spectrum of clinical presentations has been observed among carriers of triplet expansions in fragile X mental retardation 1of intermediate length. These include both mental (anxiety, depression, ADHD, and schizophrenia^28^) and neurological (migraine,^29^ late-onset tremor, and ataxia^30^) outcomes. This varied clinical presentation of an unstable mutation that can increase in severity by each generation may explain some of the familial associations observed between ASD and other mental as well as neurological diagnoses. Familiality includes genetic heritability, within-family environmental effect, and their interactions. The agreement between our findings and genetics findings may provide insight to future research on further dissecting the different components of familiality in ASD and identifying determinants of ASD susceptibility.

Autism spectrum disorders with and without ID showed different patterns of familial association. For the disorders that were associated with both, their associations with ASD with ID tended to be weaker, suggesting that family history of mental and neurological disorders was more relevant for higher-functioning ASD. Although having relatives with ASD without ID was associated with higher odds of ASD with ID in index persons and vice versa, the cross-subtype associations were not as strong as the familial recurrence risk of each subtype. In fact, beyond first-degree relatives, a child’s odds for ASD without ID did not differ in association with the presence of ID among relatives diagnosed with ASD. These findings support that ASD with and without ID share underlying mechanisms, yet each also has unique etiologic components, in good agreement with recent genetic analyses of ASD with and without ID.^22,31^ Higher familial recurrence odds of ASD with ID compared with that of ASD without ID may be due to the added effect of high heritability of both ASD and cognitive abilities.^9,32^ Despite the higher odds of familial recurrence, ASD with ID appears to be less prevalent in the population. This may be due to gaps in diagnosis, lower social and/or reproductive fitness,^33^ and other reasons that may be investigated in future research.

### Strengths and Limitations

Strengths of this study include access to a large set of high-quality family data from population-based registers. From this sample, we were able to identify interesting associations, such as the association between ASD with and without ID and cerebral palsy, that were only among first-degree relatives. A recent study reported common transcriptional patterns between cerebral palsy and ASD, primarily with genes involved in innate immunity.^34^ Also, by examining the extended family tree, we observed a consistent pattern of association across kinship distance, such that most disorders in first-degree relatives that were associated with higher odds of ASD with and without ID in index persons were also associated with the outcome in second-degree relatives. High clinical validity of the cases found in previous validation studies ensures the validity of inferences from this sample. Medical care is free for children younger than 18 years, and universal care is provided to all adults with minimal cost, decreasing potential information bias owing to differential access to health care by socioeconomic status.^35^ Ascertainment of a large number of families from population-based registers minimized selection bias. We applied false discovery rate control to the analysis to minimize the possibility of false-positive findings.

Like all epidemiologic studies, our study had some limitations. We were not able to obtain data on specific disorders that may also be relevant in the familial risk of ASD, such as tic disorders and conduct and oppositional disorders.^10^ Also, we were not able to include multiple births in this study due to a lack of zygosity information. Given the etiologic overlap and familial aggregation of the disorders under study, we did not adjust for comorbidity or family mental and neurological history in the main analysis. Adjusting for comorbidity and family history would likely have moved the OR estimates toward the null. We examined the robustness of these estimates in the sensitivity analysis adjusting for relative’s ASD status and observed the same trend of associations. However, this does not rule out the possibility that other disorders we did not include in this investigation might have played a role in the observed associations. Estimates for disorders with low prevalence among affected relatives and associations of half-relatives by relative in common (eg, maternal/paternal half-siblings) should be reexamined in future studies for confirmation.

## Conclusions

Family history of mental and neurological disorders is associated with ASD. The association may differ by the presence or absence of co-occurring ID.

## Notes

Supplement. **eTable 1.** International Classification of Diseases Codes Used for Data Extraction of Diagnoses Examined in This Study, and Age At Onset Cut Offs and Sample Sizes Used in the Sensitivity Analysis **eTable 2.** Prevalence (per 1000) of Mental and Neurological Disorders in Families With Index Persons (A) Affected vs Unaffected by Autism Spectrum Disorders; (B) Affected by Autism Spectrum Disorders With and Without Intellectual Disability **eTable 3.** Benjamini-Hochberg Adjusted P Values Associated With Odds Ratios in Table 2 **eTable 4.** Sensitivity Analysis 1: Odds Ratios Comparing Risk of Autism Spectrum Disorders With and Without Intellectual Disability in Index Persons With vs Without a Family History of Mental and Neurological Disorders, (A) Among Index Persons With Both Parents Born in Sweden; (B) Among Index Persons With At Least One Parent Not Born in Sweden **eTable 5.** Sensitivity Analysis 2: Odds Ratios Comparing Risk of Autism Spectrum Disorders With and Without Intellectual Disability in Index Persons With vs Without a Family History of Mental and Neurological Disorders, Based on Data Accounted for Potential Immortal Time Bias **eTable 6.** Sensitivity Analysis 3: Odds Ratios Comparing Risk of Autism Spectrum Disorders With and Without Intellectual Disability in Index Persons With vs Without a Family History of Mental and Neurological Disorders, Adjusting for Relative’s Autism Spectrum Disorders Status **eTable 7.** Sensitivity Analysis 4: Odds Ratios Comparing Risk of Autism Spectrum Disorders With and Without Intellectual Disability in Index Persons With vs Without a Family History of Mental and Neurological Disorders, (A) Among Index Persons Born Between 1984 and 1996; (B) Among Index Persons Born Between 1997 and 2009 **eTable 8.** Sensitivity Analysis 5: Odds Ratios Comparing Risk of Autism Spectrum Disorders With and Without Intellectual Disability in Index Persons With vs Without a Family History of Mental and Neurological Disorders, (A) Using Data From Relatives Younger Than Age 40 Years At the End of Follow-up; (B) Using Data From Relatives Age 40 Years or older At the End of Follow-up
