# Supplementary material for: Family History of Mental and Neurological Disorders and Risk of Autism
Source: JAMA Netw Open. 2019 Mar 1;2(3):e190154. doi: 10.1001/jamanetworkopen.2019.0154 (PMC6484646; doi:10.1001/jamanetworkopen.2019.0154)
Supplement: Supplement. — eTable 1. International Classification of Diseases Codes Used for Data Extraction of Diagnoses Examined in This Study, and Age At Onset Cut Offs and Sample Sizes Used in the Sensitivity Analysis eTable 2. Prevalence (per 1000) of Mental and Neurological Disorders in Families With Index Persons (A) Affected vs Unaffected by Autism Spectrum Disorders; (B) Affected by Autism Spectrum Disorders With and Without Intellectual Disability eTable 3. Benjamini-Hochberg Adjusted P Values Associated With Odds Ratios in Table 2 eTable 4. Sensitivity Analysis 1: Odds Ratios Comparing Risk of Autism Spectrum Disorders With and Without Intellectual Disability in Index Persons With vs Without a Family History of Mental and Neurological Disorders, (A) Among Index Persons With Both Parents Born in Sweden; (B) Among Index Persons With At Least One Parent Not Born in Sweden eTable 5. Sensitivity Analysis 2: Odds Ratios Comparing Risk of Autism Spectrum Disorders With and Without Intellectual Disability in Index Persons With vs Without a Family History of Mental and Neurological Disorders, Based on Data Accounted for Potential Immortal Time Bias eTable 6. Sensitivity Analysis 3: Odds Ratios Comparing Risk of Autism Spectrum Disorders With and Without Intellectual Disability in Index Persons With vs Without a Family History of Mental and Neurological Disorders, Adjusting for Relative’s Autism Spectrum Disorders Status eTable 7. Sensitivity Analysis 4: Odds Ratios Comparing Risk of Autism Spectrum Disorders With and Without Intellectual Disability in Index Persons With vs Without a Family History of Mental and Neurological Disorders, (A) Among Index Persons Born Between 1984 and 1996; (B) Among Index Persons Born Between 1997 and 2009 eTable 8. Sensitivity Analysis 5: Odds Ratios Comparing Risk of Autism Spectrum Disorders With and Without Intellectual Disability in Index Persons With vs Without a Family History of Mental and Neurological Disorders, (A) Using Data From Relatives Younger Th [file jamanetwopen-2-e190154-s001.pdf]

Supplementary Online Content

Xie S, Karlsson H, Dalman C, et al. Family history of mental and neurological disorders and risk of autism. *JAMA Netw Open*. 2019;2(3):e190154. doi:10.1001/jamanetworkopen.2019.0154

eTable 1. International Classification of Diseases Codes Used for Data Extraction of Diagnoses Examined in This Study, and Age At Onset Cut Offs and Sample Sizes Used in the Sensitivity Analysis

eTable 2. Prevalence (per 1000) of Mental and Neurological Disorders in Families With Index Persons (A) Affected vs Unaffected by Autism Spectrum Disorders; (B) Affected by Autism Spectrum Disorders With and Without Intellectual Disability

eTable 3. Benjamini-Hochberg Adjusted P Values Associated With Odds Ratios in Table 2

eTable 4. Sensitivity Analysis 1: Odds Ratios Comparing Risk of Autism Spectrum Disorders With and Without Intellectual Disability in Index Persons With vs Without a Family History of Mental and Neurological Disorders, (A) Among Index Persons With Both Parents Born in Sweden; (B) Among Index Persons With At Least One Parent Not Born in Sweden

eTable 5. Sensitivity Analysis 2: Odds Ratios Comparing Risk of Autism Spectrum Disorders With and Without Intellectual Disability in Index Persons With vs Without a Family History of Mental and Neurologic Disorders, Based on Data Accounted for Potential Immortal Time Bias

eTable 6. Sensitivity Analysis 3: Odds Ratios Comparing Risk of Autism Spectrum Disorders With and Without Intellectual Disability in Index Persons With vs Without a Family History of Mental and Neurologic Disorders, Adjusting for Relative's Autism Spectrum Disorders Status

eTable 7. Sensitivity Analysis 4: Odds Ratios Comparing Risk of Autism Spectrum Disorders With and Without Intellectual Disability in Index Persons With vs Without a Family History of Mental and Neurological Disorders, (A) Among Index Persons Born Between 1984 and 1996; (B) Among Index Persons Born Between 1997 and 2009

eTable 8. Sensitivity Analysis 5: Odds Ratios Comparing Risk of Autism Spectrum Disorders With and Without Intellectual Disability in Index Persons With vs Without a Family History of Mental and Neurological Disorders, (A) Using Data From Relatives Younger Than Age 40 Years At the End of Follow-up; (B) Using Data From Relatives Age 40 Years or older At the End of Follow-up

This supplementary material has been provided by the authors to give readers additional information about their work.

© 2019 Xie S et al. *JAMA Network Open*.

eTable 1. *International Classification of Diseases* Codes Used for Data Extraction of Diagnoses Examined in This Study, and Age At Onset Cut Offs and Sample Sizes Used in the Sensitivity Analysis

Diagnosis	ICD-10-SE	ICD-9-SE	ICD-8-SE	Age at Onset (years) Cut-Offs [a]	No. (%) Observation Excluded from Sensitivity Analysis
Mental					
ASD	F84	299	-	1	128976 (1.5) for ASD without ID; 35487 (0.4) for ASD with ID
ID	F70-79	317-319	310-315	0	4671 (0.1)
ADHD	F90	314	-	3	208240 (2.4)
Other Childhood Disorders	F80-83, 88, 89, 90-98	307.0, 307.6, 312-316	306.0-306.3, 308	0	21736 (0.3)
Alcohol Misuse	F10	291, 303, 305A	291, 303	13	1226129 (14.2)
Drug Misuse	F11-16, 18-19	292, 304, 305.C-305.X	294, 304	2	44751 (0.5)
NAPD	F20-29	295, 297, 298	295, 297, 298.3, 298.9, 299	8	629479 (7.3)
Bipolar Disorder	F30-31	296 (all except 296.2 and 296.3)	296	10	865869 (10.0)
Depression	F32-39	296.2, 296.3, 300.4, 311	300.4	11	997887 (11.5)
Anxiety Disorders	F40-41	300.0, 300.2	300.0, 300.2	6	417526 (4.8)
OCD	F42	300.3	300.3	6	421334 (4.9)
Stress Related Disorders	F43	308-309	-	3	215201 (2.5)
Other Neurotic Disorders	F44-48	300.1, 300.8, 306, 307.8	300.1, 300.5-300.7, 305	1	47527 (0.6)
Eating Disorder	F50	307.B, 307.F	306.5	12 [b]	1118150 (12.9)

© 2019 Xie S et al. *JAMA Network Open*.

Personality Disorder	F60-69	301	301	7	516483 (6.0)
Neurologic					
Cerebral Palsy	G80	343	343	0	24406 (0.3)
Epilepsy	G40-41	345	345	0	15476 (0.2)
Multiple Sclerosis	G35	340	340	14	1349655 (15.6)
Migraine	G43	346	346	4	224213 (2.6)
Dementia	F00-03	290A, 331A, 290E/W/X	290.00, 290.10, 293	24 [c]	2715016 (31.4)
Stroke	G45, I60-61, I63-I64, I69	430, 431, 434-438	430-431, 433-434	3	129578 (1.5)
Parkinson's Disease	G20	332.0	342	29	3336939 (38.6)

Abbreviations: ASD: autism spectrum disorders; ADHD: attention-deficit/hyperactivity disorder; ICD: International Classification of Diseases; ID: intellectual disability; NAPD: schizophrenia and other non-affective psychotic disorders; OCD: obsessive compulsive disorder.

a. All age at onset cut-offs were set at the 0.5th percentile (i.e., a lower bound) of age at diagnosis for each disorder in the study sample unless noted in the footnotes below.

b. The 20th percentile of age at onset was used as the cut-off for eating disorder.

c. The 5th percentile of age at onset was used as the cut-off for dementia.

© 2019 Xie S et al. *JAMA Network Open*.

eTable 2A. Prevalence (per 1000) of Mental and Neurologic Disorders in Families With Index Persons Affected vs Unaffected by Autism Spectrum Disorders

Diagnosis	IP	IP	GS 50% Relatives		GS 25% Relatives		GS 12.5% Relatives		GS 6.25% Relatives	
	IP with ASD	IP without ASD	IP with ASD	IP without ASD	IP with ASD	IP without ASD	IP with ASD	IP without ASD	IP with ASD	IP without ASD
Mental										
ASD	1000.0	0.0	132.6	20.6	47.4	19.1	103.1	64.8	72.1	55.6
ID	235.0	6.8	51.6	15.2	35.1	22.3	63.9	46.9	48.2	36.7
ADHD	411.1	26.4	158.8	44.2	85.5	47.0	177.4	126.6	146.9	127.7
Other Childhood Disorders	598.1	83.4	240.8	112.5	128.9	78.9	308.2	261.8	254.0	219.2
Alcohol Misuse	30.1	20.3	118.7	82.8	276.5	243.0	175.2	141.8	114.7	102.5
Drug Misuse	29.0	9.7	70.1	41.1	124.4	95.0	107.9	82.3	67.5	64.5
NAPD	19.8	1.8	37.9	19.7	104.4	82.1	41.7	30.4	19.0	17.9
Bipolar Disorder	16.9	2.4	41.8	20.3	85.1	66.3	35.8	30.0	25.5	19.5
Depression	125.4	29.1	395.4	241.1	482.3	424.9	282.3	233.0	194.5	162.4
Anxiety Disorders	142.4	35.0	292.3	185.6	334.2	290.6	265.8	219.0	183.1	161.4
OCD	41.9	3.6	23.4	11.8	24.5	18.1	31.3	27.6	21.2	19.8
Stress Related Disorders	59.0	21.3	338.7	217.9	305.8	271.6	211.7	172.1	138.7	119.4
Other Neurotic Disorders	14.1	3.9	48.8	30.5	88.3	74.1	44.8	35.2	28.2	23.4
Eating Disorder	30.2	11.7	34.9	25.5	31.7	27.0	77.5	62.5	52.5	45.7
Personality Disorder	28.5	4.3	86.8	35.0	104.6	75.3	80.2	58.4	61.0	45.0

© 2019 Xie S et al. *JAMA Network Open*.

Neurologic										
Cerebral Palsy	16.4	2.4	7.3	4.5	11.6	11.0	16.9	16.2	15.6	12.0
Epilepsy	70.9	8.1	46.9	32.0	137.9	119.0	68.8	64.0	57.1	46.3
Multiple Sclerosis	0.3	0.2	7.7	6.6	20.2	20.0	6.9	5.6	3.7	3.2
Migraine	23.4	17.7	121.8	97.3	141.3	127.4	119.8	106.3	81.0	73.6
Dementia	11.7	6.1	7.3	8.1	751.9	727.4	117.7	92.5	45.7	39.4
Stroke	1.5	0.8	0.8	1.3	400.9	385.0	26.6	22.2	11.7	8.7
Parkinson's Disease	0.0	0.0	0.1	0.0	38.9	38.6	1.3	0.6	0.9	0.2

Abbreviations: ASD: autism spectrum disorders; ADHD: attention-deficit/hyperactivity disorder; GS: genetic similarity; IP: index person; ID: intellectual disability; NAPD: schizophrenia and other non-affective psychotic disorders; OCD: obsessive compulsive disorder.

© 2019 Xie S et al. *JAMA Network Open*.

eTable 2B. Prevalence (per 1000) of Mental and Neurologic Disorders in Families With Index Persons Affected by Autism Spectrum Disorders With and Without Intellectual Disability

Diagnosis	IP	IP	GS 50% Relatives		GS 25% Relatives		GS 12.5% Relatives		GS 6.25% Relatives	
	IP with ASD without ID	IP with ASD with ID	IP with ASD without ID	IP with ASD with ID	IP with ASD without ID	IP with ASD with ID	IP with ASD without ID	IP with ASD with ID	IP with ASD without ID	IP with ASD with ID
Mental										
ASD	1000.0	1000.0	131.4	136.4	48.0	45.1	104.2	98.9	74.7	61.5
ID	0.0	1000.0	33.4	110.7	31.7	47.4	64.7	60.9	49.4	43.1
ADHD	434.8	334.0	166.0	135.2	89.3	72.2	179.2	170.7	150.6	132.3
Other Childhood Disorders	583.1	646.9	241.3	238.9	131.6	119.1	308.0	309.2	262.8	218.5
Alcohol Misuse	34.8	14.8	121.5	109.5	283.9	250.0	172.3	186.4	108.8	138.5
Drug Misuse	33.5	14.4	72.1	63.9	128.4	110.1	105.7	116.3	65.1	76.9
NAPD	20.5	17.5	35.9	44.4	105.2	101.5	41.6	42.4	19.2	18.5
Bipolar Disorder	20.3.	5.8	44.4	33.1	87.8	75.4	36.5	33.2	25.3	26.2
Depression	146.8	55.7	411.5	342.9	491.3	449.9	281.1	287.0	192.7	201.5
Anxiety Disorders	160.3	84.2	298.5	272.0	339.7	314.5	263.9	273.4	184.7	176.9
OCD	45.1	31.6	23.1	24.2	24.2	25.3	31.6	29.9	22.6	15.4
Stress Related Disorders	65.0	39.4	352.9	292.7	311.4	285.6	210.3	217.4	139.5	135.4

© 2019 Xie S et al. *JAMA Network Open*.

Other Neurotic Disorders	14.5	12.9	48.1	51.1	89.3	84.8	42.7.0	52.7	26.8	33.8
Eating Disorder	33.6	19.1	37.2	27.3	34.0	23.5	74.9	87.5	50.6	60.0
Personality Disorder	32.6	15.2	89.3	78.7	107.2	95.2	79.9	81.0	61.7	58.5
Neurologic										
Cerebral Palsy	5.3	52.6	6.3	10.5	10.7	14.9	18.3	11.4	16.1	13.8
Epilepsy	33.9	191.3	41.2	65.5	139.3	133.1	69.3	66.8	52.1	76.9
Multiple Sclerosis	0.0	1.2	8.4	5.5	21.2	16.7	6.9	7.1	3.8	3.1
Migraine	26.1	14.8	122.5	119.6	145.1	127.7	120.0	119.0	77.0	96.9
Dementia	9.7	18.3	140.2	176.5	758.9	727.0	113.4	134.2	42.9	56.9
Stroke	1.2	2.3	31.4	35.9	395.8	419.2	25.1	32.6	12.3	9.2
Parkinson’s Disease	0.0	0.0	2.3	2.3	38.8	39.3	1.3	1.6	0.8	1.5

Abbreviations: ASD: autism spectrum disorders; ADHD: attention-deficit/hyperactivity disorder; GS: genetic similarity; IP: index person; ID: intellectual disability; NAPD: schizophrenia and other non-affective psychotic disorders; OCD: obsessive compulsive disorder.

© 2019 Xie S et al. *JAMA Network Open*.

eTable 3. Benjamini-Hochberg Adjusted *P* Values Associated With Odds Ratios in Table 2

Diagnosis in Relatives	OR of ASD without ID in Index Persons [a]				OR of ASD with ID in Index Persons			
GS [b]	50%	25%	12.5%	6.25%	50%	25%	12.5%	6.25%
Mental								
ASD without ID	8.10×10^{-16}	8.10×10^{-16}	8.10×10^{-16}	3.68×10^{-3}	1.84×10^{-15}	6.33×10^{-10}	7.12×10^{-7}	0.81
ASD with ID	8.10×10^{-16}	6.87×10^{-7}	4.51×10^{-6}	0.29	1.84×10^{-15}	2.17×10^{-5}	0.01	0.63
ID	8.10×10^{-16}	8.10×10^{-16}	8.10×10^{-16}	7.89×10^{-3}	1.84×10^{-15}	3.05×10^{-7}	3.24×10^{-12}	0.77
ADHD	8.10×10^{-16}	2.89×10^{-5}	4.93×10^{-12}	2.81×10^{-4}	1.84×10^{-15}	1.84×10^{-15}	0.02	0.49
Other Childhood Disorders	8.10×10^{-16}	8.10×10^{-16}	8.10×10^{-16}	4.49×10^{-6}	1.84×10^{-15}	1.01×10^{-11}	3.64×10^{-11}	0.65
Alcohol Misuse	8.10×10^{-16}	1.85×10^{-15}	2.69×10^{-6}	0.39	2.29×10^{-3}	0.05	6.21×10^{-3}	0.07
Drug Misuse	8.10×10^{-16}	8.10×10^{-16}	1.56×10^{-4}	0.93	4.45×10^{-5}	2.89×10^{-3}	6.21×10^{-3}	0.48
NAPD	8.10×10^{-16}	1.08×10^{-11}	3.68×10^{-3}	0.87	7.08×10^{-12}	0.02	0.32	0.71
Bipolar Disorder	8.10×10^{-16}	4.92×10^{-11}	0.13	0.08	4.29×10^{-3}	0.07	0.42	0.83
Depression	8.10×10^{-16}	8.10×10^{-16}	3.12×10^{-11}	1.38×10^{-4}	1.84×10^{-15}	1.60×10^{-7}	0.01	0.75
Anxiety Disorders	8.10×10^{-16}	9.78×10^{-14}	3.08×10^{-11}	3.92×10^{-3}	1.84×10^{-15}	8.76×10^{-6}	0.04	0.71
OCD	8.10×10^{-16}	4.06×10^{-4}	0.25	0.35	1.36×10^{-5}	6.21×10^{-3}	0.75	0.20

© 2019 Xie S et al. *JAMA Network Open*.

Stress Related Disorders	8.10×10^{-16}	6.89×10^{-9}	8.78×10^{-7}	0.06	1.84×10^{-15}	2.67×10^{-4}	0.02	0.73
Other Neurotic Disorders	8.10×10^{-16}	8.03×10^{-5}	0.17	0.47	7.68×10^{-6}	0.04	0.02	0.65
Eating Disorder	2.18×10^{-11}	4.42×10^{-3}	3.12×10^{-3}	0.59	0.89	0.65	0.01	0.65
Personality Disorder	8.10×10^{-16}	8.10×10^{-16}	1.14×10^{-6}	1.22×10^{-4}	1.84×10^{-15}	3.13×10^{-4}	0.07	0.64
Neurologic								
Cerebral Palsy	9.68×10^{-3}	0.63	0.26	0.18	1.44×10^{-3}	0.16	0.07	0.92
Epilepsy	6.99×10^{-6}	1.80×10^{-5}	0.29	0.22	1.84×10^{-15}	0.02	0.65	0.02
Multiple Sclerosis	0.07	0.80	0.73	0.63	0.68	0.62	0.87	0.73
Migraine	8.10×10^{-16}	8.18×10^{-4}	0.15	0.96	0.02	0.34	0.74	0.47
Dementia	0.04	1.31×10^{-11}	0.11	0.63	0.62	1.00×10^{-5}	0.04	0.60
Stroke	0.27	0.09	0.52	0.12	0.44	0.01	0.71	0.71
Parkinson's Disease	0.15	0.68	0.22	0.19	-	0.75	0.47	0.31

Abbreviations: ASD: autism spectrum disorders; ADHD: attention-deficit/hyperactivity disorder; GS: genetic similarity; IP: index person; ID: intellectual disability; NAPD: schizophrenia and other non-affective psychotic disorders; OCD: obsessive compulsive disorder.

© 2019 Xie S et al. *JAMA Network Open*.

eTable 4A. Sensitivity Analysis 1: Odds Ratios Comparing Risk of Autism Spectrum Disorders With and Without Intellectual Disability in Index Persons With vs Without a Family History of Mental and Neurologic Disorders, Among Index Persons With Both Parents Born in Sweden

Diagnosis in Relatives	OR of ASD without ID in Index Persons [a]				OR of ASD with ID in Index Persons			
GS [b]	50%	25%	12.5%	6.25%	50%	25%	12.5%	6.25%
Mental								
ASD without ID	8.8 ■	2.6 ■	1.7 ■	1.3 ▪	4.8 ■	2.6 ■	1.5 ■	1.0
ASD with ID	4.1 ■	2.2 ■	1.6 ■	1.2	16.8 ■	3.0 ▪	1.5	0.7
ID	2.5 ■	1.4 ▪	1.5 ■	1.4 ▪	9.2 ■	2.5 ■	1.2	1.2
ADHD	4.7 ■	1.9 ■	1.5 ■	1.2 ▪	3.8 ■	1.8 ■	1.5 ■	1.0
Other Childhood Disorders	3.0 ■	1.7 ■	1.3 ■	1.2 ■	2.9 ■	1.7 ■	1.3 ■	1.0
Alcohol Misuse	1.4 ■	1.2 ■	1.2 ■	1.0	1.4 ■	1.1 ▪	1.2 ▪	1.2
Drug Misuse	1.9 ■	1.3 ■	1.2 ▪	1.0	1.5 ▪	1.3 ▪	1.2 ·	1.0
NAPD	1.9 ■	1.3 ■	1.2 ▪	1.0	1.9 ▪	1.2	1.1	0.7
Bipolar Disorder	2.3 ■	1.3 ■	1.1	1.3	1.5 ·	1.2	0.8	0.9
Depression	2.2 ■	1.2 ■	1.1 ■	1.2 ▪	1.6 ■	1.2 ■	1.1 ▪	0.9
Anxiety Disorders	1.9 ■	1.2 ■	1.1 ■	1.2 ▪	1.5 ■	1.2 ■	1.1	1.0
OCD	2.1 ■	1.2	1.1	1.2	2.2 ■	1.5 ·	0.9	0.6
Stress Related Disorders	1.9 ■	1.2 ■	1.1 ■	1.1	1.5 ■	1.2 ▪	1.1 ·	0.9
Other Neurotic Disorders	1.7 ■	1.2 ■	1.1	1.1	2.0 ■	1.2	1.3 ·	1.2
Eating Disorder	1.5 ■	1.2 ▪	1.1 ·	1.1	1.1	0.9	1.3 ·	1.1
Personality Disorder	2.9 ■	1.4 ■	1.5 ■	1.4 ▪	2.7 ■	1.3 ▪	1.1	1.1
Neurologic								
Cerebral Palsy	1.4	0.9	1.2	1.2	2.9 ▪	1.4	0.7	1.1
Epilepsy	1.3 ▪	1.2 ■	1.1	1.1	2.1 ■	1.2 ·	1.0	1.3 ·
Multiple Sclerosis	1.3	1.0	1.0	1.1	1.0	1.0	1.0	0.8
Migraine	1.4 ■	1.2 ■	1.1	1.0	1.1	1.2 ·	1.0	1.1

© 2019 Xie S et al. *JAMA Network Open*.

Dementia	0.8	1.1 ■	1.1	1.0	1.3	1.1 ▪	1.1	1.2
Stroke	0.7	1.0	1.0	1.4	0.5	1.1	1.1	0.6
Parkinson's Disease	- [c]	1.0	1.8	2.7	- [c]	0.9	2.2	- [c]

Abbreviations: ASD: autism spectrum disorders; ADHD: attention-deficit/hyperactivity disorder; ID: intellectual disability; NAPD: schizophrenia and other non-affective psychotic disorders; OCD: obsessive compulsive disorder; OR: odds ratio.

a. ORs with multiplicity-adjusted p-values < 0.0001, 0.0001 - < 0.01, and 0.01 - <0.05, were denoted with ■, ▪, and ·, respectively. ORs with multiplicity-adjusted p-values ≥ 0.05 were not marked.

b. GS: Genetic similarity. GS 50% relatives included fathers, mothers and full-siblings; GS 25% relatives included grandparents, uncles, aunts and half-siblings; GS 12.5% relatives included half-uncles, half-aunts and first cousins; GS 6.25% relatives included half-cousins

c. OR could not be estimated due to low event rate.

© 2019 Xie S et al. *JAMA Network Open*.

eTable 4B. Sensitivity Analysis 1: Odds Ratios Comparing Risk of Autism Spectrum Disorders With and Without Intellectual Disability in Index Persons With vs Without a Family History of Mental and Neurologic Disorders, Among Index Persons With At Least One Parent Not Born in Sweden

Diagnosis in Relatives	OR of ASD without ID in Index Persons [a]				OR of ASD with ID in Index Persons			
GS [b]	50%	25%	12.5%	6.25%	50%	25%	12.5%	6.25%
Mental								
ASD without ID	9.2 ■	2.4 ■	1.9 ■	1.2	3.6 ■	1.7	1.6	1.5
ASD with ID	3.7 ■	1.6	1.2	1.1	10.8 ■	2.4	1.7	0.8
ID	2.1 ■	1.3	1.2	1.2	5.7 ■	2.0 ▪	1.3	0.9
ADHD	4.3 ■	1.9 ■	1.5 ■	1.0	2.8 ■	1.1	1.3	1.1
Other Childhood Disorders	2.6 ■	1.6 ■	1.2 ▪	1.1	2.3 ■	1.2	1.2	0.9
Alcohol Misuse	1.7 ■	1.1	1.1	1.1	1.0	0.9	1.1	1.4
Drug Misuse	1.6 ■	1.3 ▪	1.1	1.0	1.3	1.0	1.2	1.9
NAPD	1.9 ■	1.1	1.0	0.9	1.9 ■	1.2	1.4	1.7
Bipolar Disorder	2.2 ■	1.3 ▪	1.2	1.3	1.4	1.1	1.2	1.8
Depression	1.8 ■	1.1	1.2 ·	1.1	1.2 ▪	1.1	1.0	1.7
Anxiety Disorders	1.6 ■	1.0	1.2 ▪	1.0 ▪	1.3 ▪	1.0	1.1	0.9
OCD	1.9 ▪	1.8 ▪	1.1	0.8	1.6	1.6	1.0	1.0
Stress Related Disorders	1.7 ■	1.0	1.2 ·	1.1	1.2 ▪	1.1	1.1	1.4
Other Neurotic Disorders	1.6 ■	1.1	1.1	1.1	1.1	1.1	1.3	0.7
Eating Disorder	1.5 ▪	1.1	1.3	0.9	0.9	0.9	1.2	0.9
Personality Disorder	2.2 ■	1.3 ▪	1.2	1.3	1.4	1.2	1.3	0.9
Neurologic								
Cerebral Palsy	1.7	1.2	0.9	1.5	1.4	1.3	0.4	- [c]
Epilepsy	1.3 ·	1.1	1.0	1.5	1.9 ■	1.2	0.7	2.5
Multiple Sclerosis	1.3	0.9	1.6	2.1	0.6	0.6	1.5	- [c]

© 2019 Xie S et al. *JAMA Network Open*.

Migraine	1.2 ▪	0.9	0.9	1.1	1.2	0.8	1.0	1.2
Dementia	0.6	1.1	1.0	1.5	1.0	1.1	1.2	1.0
Stroke	0.4	1.1 ·	0.8	1.0	0.6	1.2	1.1	2.0
Parkinson’s Disease	- [c]	1.0	1.2	5.4	- [c]	1.2	- [c]	- [c]

Abbreviations: ASD: autism spectrum disorders; ADHD: attention-deficit/hyperactivity disorder; ID: intellectual disability; NAPD: schizophrenia and other non-affective psychotic disorders; OCD: obsessive compulsive disorder; OR: odds ratio.

a. ORs with multiplicity-adjusted p-values < 0.0001, 0.0001 - < 0.01, and 0.01 - <0.05, were denoted with ■, ▪, and ·, respectively. ORs with multiplicity-adjusted p-values ≥ 0.05 were not marked.

b. GS: Genetic similarity. GS 50% relatives included fathers, mothers and full-siblings; GS 25% relatives included grandparents, uncles, aunts and half-siblings; GS 12.5% relatives included half-uncles, half-aunts and first cousins; GS 6.25% relatives included half-cousins

c. OR could not be estimated due to low event rate.

© 2019 Xie S et al. *JAMA Network Open*.

eTable 5. Sensitivity Analysis 2: Odds Ratios Comparing Risk of Autism Spectrum Disorders With and Without Intellectual Disability in Index Persons With vs Without a Family History of Mental and Neurologic Disorders, Based on Data Accounted for Potential Immortal Time Bias

Diagnosis in Relatives	OR of ASD without ID in Index Persons [a]				OR of ASD with ID in Index Persons			
GS [b]	50%	25%	12.5%	6.25%	50%	25%	12.5%	6.25%
Mental								
ASD without ID	9.0 ■	2.5 ■	1.8 ■	1.3 ▪	4.1 ■	2.3 ■	1.5 ■	1.0
ASD with ID	3.8 ■	2.0 ■	1.5 ■	1.2	14.2 ■	2.8 ■	1.6 ·	0.8
ID	2.3 ■	1.3 ■	1.4 ■	1.4	7.6 ■	2.4 ■	1.3 ·	1.2
ADHD	4.7 ■	1.9 ■	1.5 ■	1.1 ▪	3.3 ■	1.6 ■	1.5 ■	1.0
Other Childhood Disorders	2.9 ■	1.7 ■	1.3 ■	1.2 ■	2.6 ■	1.6 ■	1.3 ■	1.0
Alcohol Misuse	1.5 ■	1.2 ■	1.1 ■	1.1	1.2 ▪	1.1 ·	1.2 ▪	1.2
Drug Misuse	1.8 ■	1.3 ■	1.1	1.0	1.4 ■	1.2 ▪	1.2 ▪	1.1
NAPD	1.9 ■	1.3 ■	1.2 ▪	1.0	2.1 ■	1.2 ·	1.2	0.9
Bipolar Disorder	2.2 ■	1.3 ■	1.1	1.3	1.4 ▪	1.2	0.9	1.1
Depression	2.0 ■	1.2 ■	1.2 ■	1.2	1.4 ■	1.2 ■	1.1 ·	1.0
Anxiety Disorders	1.8 ■	1.2 ■	1.2 ■	1.1 ■	1.4 ■	1.2 ■	1.1 ·	1.0

© 2019 Xie S et al. *JAMA Network Open*.

OCD	2.1 ■	1.3	1.1	1.1	1.9 ■	1.5 ▪	1.0	0.6
Stress Related Disorders	1.8 ■	1.1 ■	1.1 ■	1.1	1.4 ■	1.2	1.1 ·	1.0
Other Neurotic Disorders	1.6 ■	1.2 ■	1.1	1.1	1.6 ■	1.2 ·	1.3 ·	1.1
Eating Disorder	1.5 ■	1.2	1.1 ·	1.0	1.0	0.9	1.3 ·	1.1
Personality Disorder	2.6 ■	1.4 ■	1.2 ■	1.4	2.1 ■	1.3	1.2	1.1
Neurologic								
Cerebral Palsy	1.5 ▪	0.9	1.1	1.3	2.2 ▪	1.4	0.7	1.0
Epilepsy	1.3 ■	1.1 ■	1.1	1.1	2.0 ■	1.2 ·	0.9	1.5 ·
Multiple Sclerosis	1.3	1.0	1.1	1.2	0.9	0.9	1.1	0.7
Migraine	1.3 ■	1.1	1.1	1.0	1.2 ·	1.1	1.0	1.1
Dementia	0.7	1.1 ■	1.1	1.2	1.4 ■	1.1	1.1	1.1
Stroke	0.6	1.0	1.0	1.2	0.6	1.1 ·	1.2	0.8
Parkinson's Disease	-[c]	1.0	1.7	3.8	-[c]	1.0	1.9	5.3

Abbreviations: ASD: autism spectrum disorders; ADHD: attention-deficit/hyperactivity disorder; ID: intellectual disability; NAPD: schizophrenia and other non-affective psychotic disorders; OCD: obsessive compulsive disorder; OR: odds ratio.

a. ORs with multiplicity-adjusted p-values < 0.0001, 0.0001 - < 0.01, and 0.01 - <0.05, were denoted with ■, ▪, and ·, respectively. ORs with multiplicity-adjusted p-values ≥ 0.05 were not marked.

b. GS: Genetic similarity. GS 50% relatives included fathers, mothers and full-siblings; GS 25% relatives included grandparents, uncles, aunts and half-siblings; GS 12.5% relatives included half-uncles, half-aunts and first cousins; GS 6.25% relatives included half-cousins.

c. OR could not be estimated due to low event rate.

© 2019 Xie S et al. *JAMA Network Open*.

eTable 6. Sensitivity Analysis 3: Odds Ratios Comparing Risk of Autism Spectrum Disorders With and Without Intellectual Disability in Index Persons With vs Without a Family History of Mental and Neurologic Disorders, Adjusting for Relative's Autism Spectrum Disorders Status

Diagnosis in Relatives	OR of ASD without ID in Index Persons [a]				OR of ASD with ID in Index Persons			
GS [b]	50%	25%	12.5%	6.25%	50%	25%	12.5%	6.25%
Mental								
ID	0.7 ■	1.1	1.2 ▪	1.3 ▪	3.9 ■	2.0 ■	1.1	1.2
ADHD	3.0 ■	1.7 ■	1.4 ■	1.1	2.0 ■	1.3 ▪	1.4 ■	1.0
Other Childhood Disorders	2.1 ■	1.5 ■	1.2 ■	1.2 ▪	1.9 ■	1.4 ■	1.2 ■	1.0
Alcohol Misuse	1.5 ■	1.2 ■	1.1 ■	1.1	1.2 ·	1.1	1.2 ·	1.2
Drug Misuse	1.7 ■	1.3 ■	1.1 ▪	1.0	1.3 ▪	1.2 ▪	1.2 ·	1.1
NAPD	1.6 ■	1.2 ■	1.1	1.0	1.8 ■	1.2	1.1	0.9
Bipolar Disorder	2.0 ■	1.3 ■	1.1	1.3	1.3	1.2	0.8	1.1
Depression	1.9 ■	1.2 ■	1.1 ■	1.2 ▪	1.3 ■	1.2 ■	1.1	1.0
Anxiety Disorders	1.6 ■	1.1 ■	1.1 ■	1.1 ·	1.3 ■	1.2 ■	1.1	1.0
OCD	1.4 ■	1.2 ·	1.0	1.1	1.3	1.4 ·	0.9	0.6
Stress Related Disorders	1.8 ■	1.1 ■	1.1 ■	1.1	1.3 ■	1.2 ▪	1.1	1.0
Other Neurotic Disorders	1.5 ■	1.2 ▪	1.1	1.1	1.5 ▪	1.2	1.3 ·	1.1
Eating Disorder	1.4 ■	1.2	1.1 ·	1.0	0.9	0.9	1.2 ·	1.1
Personality Disorder	2.2 ■	1.3 ■	1.2 ▪	1.4 ▪	1.8 ■	1.3 ▪	1.1	1.1
Neurologic								
Cerebral Palsy	1.1	0.9	1.1	1.3	1.6	1.3	0.6	1.0
Epilepsy	1.0	1.1 ▪	1.0	1.1	1.6 ■	1.1 ·	0.9	1.5 ·
Multiple Sclerosis	1.3	1.0	1.1	1.2	0.9	0.9	1.1	0.7
Migraine	1.3 ■	1.1 ▪	1.1	1.0	1.2 ·	1.1	1.0	1.1
Dementia	0.7 ·	1.1 ■	1.1	1.2	1.4	1.1 ■	1.1	1.1
Stroke	0.6	1.0	1.0	1.2	0.5	1.1 ·	1.1	0.8

© 2019 Xie S et al. *JAMA Network Open*.

Parkinson's Disease	7.6	0.9	1.8	3.8	- [c]	0.9	1.9	5.3

Abbreviations: ASD: autism spectrum disorders; ADHD: attention-deficit/hyperactivity disorder; ID: intellectual disability; NAPD: schizophrenia and other non-affective psychotic disorders; OCD: obsessive compulsive disorder; OR: odds ratio.
a. ORs with multiplicity-adjusted p-values < 0.0001, 0.0001 - < 0.01, and 0.01 - <0.05, were denoted with ■, ▪, and ·, respectively. ORs with multiplicity-adjusted p-values ≥ 0.05 were not marked.
b. GS: Genetic similarity. GS 50% relatives included fathers, mothers and full-siblings; GS 25% relatives included grandparents, uncles, aunts and half-siblings; GS 12.5% relatives included half-uncles, half-aunts and first cousins; GS 6.25% relatives included half-cousins.
c. OR could not be estimated due to low event rate.

© 2019 Xie S et al. *JAMA Network Open*.

eTable 7A. Sensitivity Analysis 4: Odds Ratios Comparing Risk of Autism Spectrum Disorders With and Without Intellectual Disability in Index Persons With vs Without a Family History of Mental and Neurologic Disorders, Among Index Persons Born Between 1984 and 1996

Diagnosis in Relatives	OR of ASD without ID in Index Persons [a]				OR of ASD with ID in Index Persons			
GS [b]	50%	25%	12.5%	6.25%	50%	25%	12.5%	6.25%
Mental								
ASD without ID	8.2 ■	2.7 ■	1.7 ■	1.2	3.7 ■	2.3 ■	1.6 ■	1.0
ASD with ID	3.5 ■	2.1 ▪	1.2	1.4	12.3 ■	2.7 ▪	1.4	0.9
ID	2.1 ■	1.4 ▪	1.3 ■	1.4 ▪	6.9 ■	2.2 ■	1.2	1.3
ADHD	4.0 ■	1.9 ■	1.5 ■	2.1	3.2 ■	1.6 ■	1.5 ■	1.1
Other Childhood Disorders	2.9 ■	1.8 ■	1.3 ■	1.1 ▪	2.6 ■	1.6 ■	1.3 ■	0.9
Alcohol Misuse	1.5 ■	1.1 ▪	1.1 ▪	1.0	1.2 ·	1.1	1.1	1.2
Drug Misuse	1.7 ■	1.2 ■	1.2 ▪	1.0	1.4 ▪	1.2 ·	1.2	1.2
NAPD	2.0 ■	1.3 ■	1.2 ▪	1.4	2.1 ■	1.3 ·	1.2	0.9
Bipolar Disorder	2.1 ■	1.3 ■	1.2	1.4 ·	1.4 ·	1.2	0.9	1.2
Depression	2.0 ■	1.2 ■	1.2 ■	1.2 ▪	1.4 ■	1.2 ▪	1.2 ▪	1.0
Anxiety Disorders	1.7 ■	1.2 ■	1.1 ■	1.1 ·	1.4 ■	1.2 ▪	1.1 ·	1.0.
OCD	2.1 ■	1.3 ·	1.1	0.8	2.3 ■	1.8 ▪	1.0	0.5
Stress Related Disorders	1.7 ■	1.1 ▪	1.1 ■	1.1	1.3 ■	1.1	1.2 ▪	0.9
Other Neurotic Disorders	1.5 ■	1.2 ▪	1.0	1.3	1.7 ■	1.2	1.4 ▪	1.3
Eating Disorder	1.5 ■	1.3 ·	1.1 ·	1.0	1.2	0.9	1.3 ▪	1.1
Personality Disorder	2.5 ■	1.3 ■	1.2 ■	1.4 ▪	2.1 ■	1.3 ▪	1.3 ·	1.2
Neurologic								
Cerebral Palsy	1.8 ▪	1.0	1.2	1.4	2.5 ▪	1.1	0.6	0.5
Epilepsy	1.2 ▪	1.1 ▪	1.0	1.1	2.1 ■	1.2 ·	1.0	1.6 ·
Multiple Sclerosis	1.4	0.9	1.0	1.2	0.8	0.7	1.3	0.9
Migraine	1.3 ■	1.1	1.0	1.1	1.1	1.2	1.1	1.2

© 2019 Xie S et al. *JAMA Network Open*.

Dementia	0.7	1.0 ·	1.0	1.1	1.2	1.1 ·	1.2	1.0
Stroke	0.5	1.0 ·	0.9	1.3	- [c]	1.1	1.0	0.6
Parkinson's Disease	13.0	0.9	1.5	6.6	- [c]	0.9	2.3	- [c]

Abbreviations: ASD: autism spectrum disorders; ADHD: attention-deficit/hyperactivity disorder; ID: intellectual disability; NAPD: schizophrenia and other non-affective psychotic disorders; OCD: obsessive compulsive disorder; OR: odds ratio.

a. ORs with multiplicity-adjusted p-values < 0.0001, 0.0001 - < 0.01, and 0.01 - <0.05, were denoted with ■, ▪, and ·, respectively. ORs with multiplicity-adjusted p-values ≥ 0.05 were not marked.

b. GS: Genetic similarity. GS 50% relatives included fathers, mothers and full-siblings; GS 25% relatives included grandparents, uncles, aunts and half-siblings; GS 12.5% relatives included half-uncles, half-aunts and first cousins; GS 6.25% relatives included half-cousins

c. OR could not be estimated due to low event rate.

© 2019 Xie S et al. *JAMA Network Open*.

eTable 7B. Sensitivity Analysis 4: Odds Ratios Comparing Risk of Autism Spectrum Disorders With and Without Intellectual Disability in Index Persons With vs Without a Family History of Mental and Neurologic Disorders, Among Index Persons Born Between 1997 and 2009

Diagnosis in Relatives	OR of ASD without ID in Index Persons [a]				OR of ASD with ID in Index Persons			
GS [b]	50%	25%	12.5 %	6.25%	50%	25%	12.5%	6.25%
Mental								
ASD without ID	9.0 ■	2.4 ■	1.8 ■	1.4 ▪	4.0 ■	2.2 ▪	1.4	1.0.
ASD with ID	3.8 ■	2.1 ▪	1.7 ■	10	15.7 ■	3.2 ▪	1.7	0.4
ID	2.3 ■	1.3 ·	1.4 ■	1.4 ·	8.1 ■	2.8 ■	1.3	1.0
ADHD	5.0 ■	2.0 ■	1.5 ■	1.2 ▪	2.9 ■	1.5 ·	1.2	0.9
Other Childhood Disorders	2.6 ■	1.7 ■	1.3 ■	1.2 ▪	2.3 ■	1.5 ▪	1.2 ▪	1.0
Alcohol Misuse	1.7 ■	1.2 ■	1.2 ▪	1.1	1.4 ·	1.0.	1.3 ·	1.1
Drug Misuse	2.0 ■	1.4 ■	1.1	1.0	1.4	1.2	1.2	1.0
NAPD	1.5 ▪	1.2 ▪	1.1	1.1	2.0 ▪	1.1	0.9	0.7
Bipolar Disorder	2.4 ■	1.3 ■	1.0	1.0	1.6	1.2	0.9	0.6
Depression	2.1 ■	1.2 ■	1.1 ·	1.1	1.4 ■	1.2 ▪	1.0	1.0
Anxiety Disorders	1.9 ■	1.1 ■	1.2 ■	1.1	1.4 ■	1.2 ·	1.0	0.9
OCD	2.2 ■	1.4 ▪	1.1	1.6 ·	1.3	1.2	0.9	0.8
Stress Related Disorders	1.8 ■	1.2 ■	1.1 ▪	1.2	1.4 ■	1.3 ▪	0.9	1.0
Other Neurotic Disorders	1.6 ■	1.2 ·	1.2	0.7	1.2	1.1	1.0	0.9
Eating Disorder	1.7 ■	1.2 ·	1.1	1.1	0.7	1.0	1.0	1.0
Personality Disorder	3.0 ■	1.5 ■	1.2 ▪	1.3	1.9 ■	1.3	1.0	0.9
Neurologic								
Cerebral Palsy	1.1	0.8	1.0	1.1	1.6	1.8	0.7	2.1
Epilepsy	1.4 ▪	1.2 ▪	1.1	1.2	2.0 ■	1.1	0.9	1.3
Multiple Sclerosis	1.2	1.2	1.2	1.2	1.0	1.2	0.3	- [c]

© 2019 Xie S et al. *JAMA Network Open*.

Migraine	1.3 ■	1.1 ▪	1.1 ·	0.9	1.2	1.0	0.7	1.0
Dementia	0.9	1.1 ■	1.1	1.0	1.3	1.2 ▪	1.1	1.5
Stroke	1.1	1.0	1.0	1.6	2.5	1.2 ·	1.3	1.5
Parkinson's Disease	- [c]	- [c]	- [c]	- [c]	- [c]	- [c]	- [c]	- [c]

Abbreviations: ASD: autism spectrum disorders; ADHD: attention-deficit/hyperactivity disorder; ID: intellectual disability; NAPD: schizophrenia and other non-affective psychotic disorders; OCD: obsessive compulsive disorder; OR: odds ratio.

a. ORs with multiplicity-adjusted p-values < 0.0001, 0.0001 - < 0.01, and 0.01 - <0.05, were denoted with ■, ▪, and ·, respectively. ORs with multiplicity-adjusted p-values ≥ 0.05 were not marked.

b. GS: Genetic similarity. GS 50% relatives included fathers, mothers and full-siblings; GS 25% relatives included grandparents, uncles, aunts and half-siblings; GS 12.5% relatives included half-uncles, half-aunts and first cousins; GS 6.25% relatives included half-cousins

c. OR could not be estimated due to low event rate.

© 2019 Xie S et al. *JAMA Network Open*.

eTable 8A. Sensitivity Analysis 5: Odds Ratios Comparing Risk of Autism Spectrum Disorders With and Without Intellectual Disability in Index Persons With vs Without a Family History of Mental and Neurologic Disorders, Using Data From Relatives Younger Than Age 40 Years At the End of Follow-up

Diagnosis in Relatives	OR of ASD without ID in Index Persons [a]				OR of ASD with ID in Index Persons			
GS [b]	50%	25%	12.5%	6.25%	50%	25%	12.5%	6.25%
Mental								
ASD without ID	8.1 ■	2.5 ■	1.8 ■	1.3 ▪	3.7 ■	2.3 ■	1.5 ■	1.0
ASD with ID	3.7 ■	2.1 ■	1.5 ■	1.2	13.6 ■	2.9 ■	1.4 ·	0.8
ID	2.2 ■	1.4 ■	1.4 ■	1.4 ▪	7.5 ■	2.9 ■	1.3 ·	1.2
ADHD	2.7 ■	1.6 ■	1.3 ■	1.2 ▪	3.1 ■	1.6 ■	1.5 ■	1.0
Other Childhood Disorders	2.7 ■	1.6 ■	1.3 ■	1.2 ■	2.4 ■	1.5 ■	1.3 ■	1.0
Alcohol Misuse	1.4 ■	1.2 ■	1.2 ■	1.0	1.0	1.3 ·	1.1	1.3 ·
Drug Misuse	1.9 ■	1.2 ▪	1.1 ▪	1.0	1.3	1.5 ▪	1.1	1.1
NAPD	2.1 ■	1.2	1.1	0.9	2.0 ▪	1.5	1.1	0.6
Bipolar Disorder	2.6 ■	1.4 ▪	1.0	1.2	1.7 ·	1.4	1.0	0.9
Depression	2.2 ■	1.4 ■	1.2 ■	1.2 ▪	1.4 ■	1.4 ■	1.1 ▪	1.1
Anxiety Disorders	2.0 ■	1.2 ■	1.2 ■	1.1 ▪	1.4 ■	1.4 ■	1.1 ·	1.0
OCD	2.1 ■	1.4 ▪	1.1	1.2	2.0 ▪	1.7 ·	0.9	0.6
Stress Related Disorders	1.9 ■	1.2 ▪	1.2 ■	1.1	1.4 ■	1.3 ▪	1.2 ·	1.0
Other Neurotic Disorders	1.8 ■	1.4 ▪	1.1	1.1	1.5 ·	1.5	1.2	1.3
Eating Disorder	1.4 ■	1.3 ▪	1.2 ▪	1.0	0.9	1.0	1.2 ·	1.1
Personality Disorder	2.6 ■	1.6 ■	1.3 ■	1.4 ▪	1.8 ■	1.5 ▪	1.1	1.1
Neurologic								
Cerebral Palsy	1.6 ▪	1.1	1.1	1.2	2.2 ▪	0.7	0.7	0.9
Epilepsy	1.4 ▪	1.2 ·	1.0	1.1	2.5 ■	1.7 ▪	0.9	1.5 ·
Multiple Sclerosis	1.4	1.2	1.2	1.1	2.2	1.9	1.1	0.6
Migraine	1.4 ■	1.2 ▪	1.0	1.0	1.1	1.0	1.0	1.2

© 2019 Xie S et al. *JAMA Network Open*.

Dementia	0.7 ·	1.3 ▪	1.0	1.0	1.1	1.2	1.2	1.4
Stroke	0.7	0.9	0.8	1.4	0.5	1.3	0.7	1.0
Parkinson’s Disease	- [c]	- [c]	- [c]	4.2	- [c]	- [c]	- [c]	- [c]

Abbreviations: ASD: autism spectrum disorders; ADHD: attention-deficit/hyperactivity disorder; ID: intellectual disability; NAPD: schizophrenia and other non-affective psychotic disorders; OCD: obsessive compulsive disorder; OR: odds ratio.

a. ORs with multiplicity-adjusted p-values < 0.0001, 0.0001 - < 0.01, and 0.01 - <0.05, were denoted with ■, ▪, and ·, respectively. ORs with multiplicity-adjusted p-values ≥ 0.05 were not marked.

b. GS: Genetic similarity. GS 50% relatives included fathers, mothers and full-siblings; GS 25% relatives included grandparents, uncles, aunts and half-siblings; GS 12.5% relatives included half-uncles, half-aunts and first cousins; GS 6.25% relatives included half-cousins

c. OR could not be estimated due to low event rate.

© 2019 Xie S et al. *JAMA Network Open*.

eTable 8B. Sensitivity Analysis 5: Odds Ratios Comparing Risk of Autism Spectrum Disorders With and Without Intellectual Disability in Index Persons With vs Without a Family History of Mental and Neurologic Disorders, Using Data From Relatives Age 40 Years or older At the End of Follow-up

Diagnosis in Relatives	OR of ASD without ID in Index Persons [a]				OR of ASD with ID in Index Persons			
GS [b]	50%	25%	12.5%	6.25%	50%	25%	12.5%	6.25%
Mental								
ASD without ID	17.0 ■	2.2 ■	1.5	- [c]	3.7 ■	2.3	1.5	1.0
ASD with ID	3.7	2.1	1.5	1.2	13.6	2.9	1.4 ·	0.8
ADHD	5.2 ■	1.7 ■	1.5 ▪	1.0	3.1 ■	1.6	1.5	1.0
ID	2.6 ■	1.2	1.3	1.8	7.5 ■	2.9	1.3	1.2
Other Childhood Disorders	4.4 ■	1.6 ■	1.6 ■	1.1	2.4 ■	1.5	1.3	1.0
Alcohol Misuse	1.6 ■	1.2 ■	1.1	1.1	1.0 ▪	1.3	1.1 ·	1.3
Drug Misuse	1.8 ■	1.4 ■	1.1	1.1	1.3 ■	1.5	1.1 ▪	1.1
NAPD	1.8 ■	1.3 ■	1.3 ▪	1.5	2.0 ■	1.5	1.1	0.6
Bipolar Disorder	2.1 ■	1.3 ■	1.3 ·	1.5	1.7	1.4	1.0	0.9
Depression	2.0 ■	1.2 ■	1.1	1.0	1.4 ■	1.4 ▪	1.1	1.1
Anxiety Disorders	1.7 ■	1.1 ■	1.1 ·	1.1	1.4 ■	1.4 ·	1.1	1.0
OCD	2.1 ■	1.3 ·	1.0	- [c]	2.0 ·	1.7	0.9	0.6
Stress Related Disorders	1.7 ■	1.1 ■	1.0	1.1	1.4 ■	1.3 ·	1.2	1.0
Other Neurotic Disorders	1.5 ■	1.1 ▪	1.1	0.9	1.5 ▪	1.5	1.2 ·	1.3
Eating Disorder	1.8 ■	1.1	0.7	1.1	0.9	1.0	1.2	1.1
Personality Disorder	2.7 ■	1.3 ■	1.1	1.2	1.8 ■	1.5 ·	1.1	1.1
Neurologic								
Cerebral Palsy	1.1	0.8	1.3	1.9	2.2	0.7 ·	0.7 ·	0.9
Epilepsy	1.3 ▪	1.1 ▪	1.1	1.0	2.5 ▪	1.7	0.9	1.5
Multiple Sclerosis	1.3	1.0	0.9	1.5	2.2	1.9	1.1	0.6

© 2019 Xie S et al. *JAMA Network Open*.

Migraine	1.3 ■	1.1 ˑ	1.1	1.1	1.1 ▪	1.0	1.0	1.2
Dementia	1.1	1.1 ■	1.1 ▪	1.2	1.1	1.2 ▪	1.2	1.4
Stroke	- [c]	1.0	1.1	1.3	- [c]	1.3	0.7	1.0
Parkinson's Disease	- [c]	1.0	2.1	3.1	- [c]	- [c]	- [c]	- [c]

Abbreviations: ASD: autism spectrum disorders; ADHD: attention-deficit/hyperactivity disorder; ID: intellectual disability; NAPD: schizophrenia and other non-affective psychotic disorders; OCD: obsessive compulsive disorder; OR: odds ratio.

a. ORs with multiplicity-adjusted p-values < 0.0001, 0.0001 - < 0.01, and 0.01 - <0.05, were denoted with ■, ▪, and ˑ, respectively. ORs with multiplicity-adjusted p-values ≥ 0.05 were not marked.

b. GS: Genetic similarity. GS 50% relatives included fathers, mothers and full-siblings; GS 25% relatives included grandparents, uncles, aunts and half-siblings; GS 12.5% relatives included half-uncles, half-aunts and first cousins; GS 6.25% relatives included half-cousins

c. OR could not be estimated due to low event rate.

© 2019 Xie S et al. *JAMA Network Open*.
